# A Novel Liquid Medium for the Efficient Growth of the Salmonid Pathogen *Piscirickettsia salmonis* and Optimization of Culture Conditions

**DOI:** 10.1371/journal.pone.0071830

**Published:** 2013-09-05

**Authors:** Mirtha Henríquez, Ernesto González, Sergio H. Marshall, Vitalia Henríquez, Fernando A. Gómez, Irene Martínez, Claudia Altamirano

**Affiliations:** 1 Escuela de Ingeniería Bioquímica, Pontificia Universidad Católica de Valparaíso, Valparaíso, Chile; 2 Laboratorio de Genética e Inmunología Molecular, Instituto de Biología, Facultad de Ciencias, Pontificia Universidad Católica de Valparaíso, Valparaíso, Chile; 3 NBC, Núcleo de Biotecnología Curauma, Curauma, Valparaíso, Chile; 4 CREAS CONICYT-REGIONAL, GORE Región de Valparaíso, Chile; Northeast Agricultural University, China

## Abstract

*Piscirickettsia salmonis* is the bacterium that causes Piscirickettsiosis, a systemic disease of salmonid fish responsible for significant economic losses within the aquaculture industry worldwide. The growth of the bacterium for vaccine formulation has been traditionally accomplished by infecting eukaryotic cell lines, a process that involves high production costs and is time-consuming. Recent research has demonstrated that it is possible to culture pure *P. salmonis* in a blood containing (cell-free) medium.

In the present work we demonstrate the growth of *P. salmonis* in a liquid medium free from blood and serum components, thus establishing a novel and simplified bacteriological medium. Additionally, the new media reported provides improved growth conditions for *P. salmonis*, where biomass concentrations of approximately 800 mg cell dry weight L^−1^ were obtained, about eight times higher than those reported for the blood containing medium. A 2- level full factorial design was employed to evaluate the significance of the main medium components on cell growth and an optimal temperature range of 23–27°C was determined for the microorganism to grow in the novel liquid media.

Therefore, these results represent a breakthrough regarding *P. salmonis* research in order to optimize pure *P. salmonis* growth in liquid blood and serum free medium.

## Introduction

*Piscirickettsia salmonis* is a fish pathogen known to cause Piscirickettsiosis, a systemic disease that affects a wide range of salmonid species and other fish species [1], [2], [3]. *P. salmonis* is a Gram-negative bacterium described as non-motile, not encapsulated, pleomorphic but generally coccoid, with a diameter ranging from 0.1 to 1.5 µm [4], [5] and is considered as facultative intracellular organism [6], [7], [8]. Nowadays, the prophylactic treatments available for controlling the disease are vaccines based on either recombinant antigens or bacterins (attenuated bacteria), which are administered through injection or oral immunization [9]. In order to produce these bacterins, high cell densities are required, which until today have been achieved by infecting eukaryotic cell lines. However, the efficiency and purity of these putative vaccines have shown variable results [10]. Consequently, there is an urgent need and interest to achieve pure high cell density growth of *P. salmonis* in a liquid medium free from eukaryotic contaminants. Moreover, *in vitro* cultivation of the microorganism in cell lines is inherently time-consuming and expensive in terms of costs. Hence, the ability to grow high densities of pure *P. salmonis* in liquid media would make feasible the reduction of production costs by avoiding the necessity of maintaining cell lines, lowering medium costs, and reducing timelines within upstream and downstream phases.

For a long time *P. salmonis* was considered an obligate intracellular pathogen and therefore eukaryotic cell lines susceptible to infection, such as Chinook salmon embryo (CHSE-214) [11] and insect tissue cells (Sf21) [12] has been used for its propagation. Nowadays is possible obtain the growth of the bacterium on solid and liquid media free from eukaryotic host cells [6], [7], [8], [13]. The range of temperature for the bacterium cultivation has been reported between 16° and 23°C, but the optimum culture temperature only has been reported as 22°C for agar cultures [7]. Currently there are three studies that describe growth of the bacterium in liquid media and the growth only have been performed at 17° and 23°C [8], [13], [14], but no studies exist regarding the optimum growth temperature in liquid media. Considering that culture conditions affect cell growth [15] and intra- and extracellular metabolites production [16], media composition and culture temperatures were evaluated in the present work in order to develop a process to produce *P. salmonis* biomass. The results of the present work represent a breakthrough concerning the culture of *P. salmonis* in liquid media by achieving growth of the bacterium for the first time at high densities, identifying potential variables for biomass optimization as well as an optimum temperature range for growth of *P. salmonis*.

## Materials and Methods

### Strain and media

*Piscirickettsia salmonis* LF-89 (ATCC VR-1361), was cultivated in different culture media as described in Table 1, with the following basal medium (BM) composition: yeast extract (Merck) 2.0 g L^−1^, peptone from meat (peptic digested, Merck) 2.0 g L^−1^, (NH_4_)_2_SO_4_ 1.32 g L^−1^, MgSO_4_·7H_2_O 0.1 g L^−1^, K_2_HPO_4_ 6.3 g L^−1^, NaCl 9.0 g L^−1^, CaCl_2_·2H_2_O 0.08 g L^−1^ and FeSO_4_·7H_2_O 0.02 g L^−1^.

**Table 1:** Media description.

Medium	Composition
BM1	BM^a^+5 g L^−1^ glucose
BM2	BM^a^+2 g L^−1^ glutamic acid
BM3	BM2 without (NH_4_)_2_SO_4_

a BM composition: yeast extract (Merck) 2.0 g L^−1^, peptone from meat (peptic digested, Merck) 2.0 g L^−1^, (NH_4_)_2_SO_4_ 1.32 g L^−1^, MgSO_4_·7H_2_O 0.1 g L^−1^, K_2_HPO_4_ 6.3 g L^−1^, NaCl 9.0 g L^−1^, CaCl_2_·2H_2_O 0.08 g L^−1^ and FeSO_4_·7H_2_O 0.02 g L^−1^.

All media components were sterilized for 15 minutes at 121°C. CaCl_2_·2H_2_O and FeSO_4_·7H_2_O were sterilized separately to avoid appearance of precipitate, and yeast extract and peptone were sterilized separately from glucose to avoid Maillard reactions.

### Culture conditions

All cultures were carried out in an orbital shaker (New Brunswick, C-24 Incubator Shaker) at 100 rpm in 250 mL Erlenmeyer flasks with a working volume of 25–50 mL. The initial medium pH was adjusted to 6.6 by addition of 1N H_2_SO_4_ in all experiences unless anything else is stated.

a) *P. salmonis growth in blood free liquid medium*

The ability of *P. salmonis* to grow in blood free liquid medium was assessed at 19°C in BM1 (see Table 1) medium at an initial pH 6.6 and 7.1.

b) *Culture temperature*

The effect of the culture temperature in cell growth was assessed by culturing *P. salmonis* in BM1 medium at 17, 19, 21, 23, 25, 27 and 29°C. Cultures were done in triplicate.

c) *Main medium components*

A main medium components screening was performed to evaluate *P. salmonis* growth in basal medium (BM) and BM supplemented with glucose (BM1) or glutamic acid (BM2), as well as in a medium without ammonium sulphate (BM3). Media composition is shown in Table 1.

### Factorial design of experiments

A 2-level full factorial design of experiments was performed to determine the significance of yeast extract (Bacto) and peptone (Merk) concentrations on cell growth according to the design matrix shown in Table 2. All the experiments were performed at 23°C. The formulation that produces the high volumetric productivity (Qx) was selected and named MB4.

**Table 2:** Design matrix: Final yeast extract and peptone concentrations in BM3.

Run	Yeast extract [g L^−1^]	Peptone [g L^−1^]
1	8	8
2	8	4
3	4	8
4	4	4
CP1^a^	6	6
CP2^a^	6	6
CP3^a^	6	6

a The centre point (CP) experience was performed in triplicate in order to estimate the experimental error of the system.

### Inoculum preparation

Initial blood and serum free liquid medium experiments and the culture temperature study were inoculated with CHSE-214 *P. salmonis*-infected culture supernatant [8] or from liquid subcultures grown in BM1 (see Table 1).

The inoculum for the main medium components and the factorial design experiments were taken from propagation flasks inoculated directly from freshly thawed *P. salmonis* infected supernatant maintained at −80°C. For the main medium components experiments (BM1, BM2 or BM3, see Table 1) propagation flasks were used in accordance with the respective medium used in the experiment. For the factorial design experiments, BM2 was used as propagation medium.

### Analytical methods

Cell growth was monitored spectrophotometrically (Spectronic 20 Genesys) by optical density (OD) measurements at 600 nm after appropriate dilution. The OD_600_ values were later correlated with biomass concentration by a calibration curve based on cell dry weight (CDW). The ammonia concentration within the cultures was measured by the spectrophotometric method described by Kaplan [17].

### Statistical analysis

Minitab® 15 Statistical Software was employed to perform an analysis of variance (ANOVA) of the factorial design in order to determine the significance (p<0.05) of the evaluated factors on biomass production.

### Cell culture infection with *P. salmonis* from liquid cultures

In order to determine if the *P. salmonis* infective potential was affected by its growth in cell free media, infection experiments were performed in two different cell lines, CHSE 214 salmon embryo cell line (ATCC CRL-1681) and the trout macrophage-monocyte RTS11 cell line [18] (kindly donated by Dr. Niels Bols; University of Waterloo, Canada). Monolayers of CHSE-214 cells were routinely propagated at 17°C in 25 cm^2^ culture flasks containing minimal essential medium (MEM, Gibco), supplemented with 7.5% fetal bovine serum (FBS) and adjusted to pH 7.2 with 10 mM sodium bicarbonate and 15 mM HEPES [5]. Monolayers/suspensions of RTS11 cells were grown at 20°C in 25 cm^2^ culture flasks containing Leibovitz L-15 media (Gibco) pH 7.3 for its propagation, supplemented with 15% FBS as has been described previously [19].

For the cell lines infection a *P. salmonis* culture in BM4 media was prepared and incubated at 23°C at 100 rpm until the culture reach an OD_600_ of 0.3. Before the infection, the *P. salmonis* cultures were tested for contamination using the Fluorotest Kit (Bioschile), according manufacturer's instructios. Then 200 µl of the previous culture were used to infect each cell line. CHSE-214 cells were monitored for Cytopathic Effect (CPE) every 48 hours for 10 days. Due to the fact that RTS11 cell line do not produce a typical CPE with *P. salmonis* infection an immunofluorescence analyses was performed 5 days post-infection, using the kit Direct SRS Fluorotest (BiosChile) according manufacturer's instructions. All cells were observed in an inverted microscopy Nikon Eclipse Ti using the 40× objective and all images were digitalized with the Nikon's NIS-Elements software.

## Results and Discussion

### *P. salmonis* growth in blood and serum free liquid medium

The capability of *P. salmonis* to grow in a typical blood free bacteriological medium was evaluated using BM1 medium (Table 1) at two different initial pH values: 6.6 and 7.1. The microorganism showed significant cell growth under both conditions, with similar growth patterns, comparable maximum specific growth rates of around 0.05 h^−1^ and final biomass concentration of approximately 0.65 OD_600_ after 115 h. Therefore, *P. salmonis* growth was successfully achieved with a simple bacteriological liquid medium.

The pH range tested was similar to the reported culture pH values for *Legionella pneumophila*, an intracellular pathogen phylogenetically related to *P. salmonis* [20]. For this bacterium, cell growth has been reported at pH 6.9 [21], [22], [23], with a maximum specific growth rate of 0.086 h^−1^ (doubling time of 8 h) at pH 6.5 [22]. As mentioned earlier, the initial pH values used in this study were similar to the ones reported for *L. pneumophila*; however, no significant differences were noticed in cell growth with respect to culture pH under the conditions tested. The higher maximum specific growth rate for *P. salmonis* was similar as it was described for *L. pneumophila*.

Generally, a decreasing pH is observed in microbial culture processes, caused by the liberation of protons due to nitrogen assimilation from ammonia or by- product formation (e.g. organic acids) or both. Interestingly, an increase in medium pH was recorded at the end of our experiments. This increment in pH could be a consequence of the degradation of amino acids present in the peptone and yeast extract as well as to the assimilation of organic acids present in them.

As no significant growth differences were found at both pH values, the use of initial pH 6.6 was selected for all other experiments to avoid an excessive increase in pH during the culture, which could negatively affect cell growth.

The culture success mentioned above is considered of great importance because not only we have accomplished to exceed the biomass concentration value in time reported for *P. salmonis* in liquid medium (0.25 OD_600_ after 13 days and 1.7 OD_600_ after 6 days) [8], [13], but also to achieve this in a simpler and rather common bacteriological medium.

### Optimal Culture temperature

*P. salmonis* cultures in BM1 medium (pH 6.6) were incubated at different temperatures in order to evaluate the optimum growth temperature of *P. salmonis* in liquid medium. The maximum specific growth rates (μ_max_) with the respective standard deviations (SD) for each studied temperature are presented in Table 3.

**Table 3:** Culture temperature impact on specific growth rate (μ_max_).

Temperature [°C]	μ_max_ a [h^−1^]
17	0.029±0.002
19	0.042±0.005
21	0.059±0.006
23	0.099±0.008
25	0.118±0.010
27	0.103±0.006
29	0.046±0.001

a Average value of replicates.

The results obtained from this study show that *P. salmonis* could be grown between 17 and 29°C, being in accordance with previous studies, where growth of *P. salmonis* in liquid media was reported at 17 and 23°C [8], [7], [14].

The highest specific growth rate (0.118 h^−1^) was recorded at 25°C, a value four times higher than the recorded at 17°C. Moreover, considering the calculated SD, an optimum temperature range for growth of *P. salmonis* between 23 and 27°C was identified.

### Main medium components

Experiments were performed with the purpose of evaluating *P. salmonis* growth in liquid medium with and without glucose addition (BM1 and BM, respectively) as well as in medium supplemented with glutamic acid (BM2). The obtained growth profiles were comparable in BM and BM1 media, reaching biomass concentrations around 0.6±0.02 OD_600_ after approximately 40 h. A slightly higher final biomass concentration was seen in the experiences carried out with glutamic acid, which reached 0.7±0.02 OD_600_ after 40 h. Additionally, the culture performed with glutamic acid presented a higher maximum specific growth rate (0.13±0.001 h^−1^) than the recorded for the experiences performed with and without glucose (0.11±0.005 h^−1^). Based on the presented results it was decided to continue the following studies with BM2 medium.

Since an increment in culture pH had been recorded in earlier experiences, it was decided to evaluate *P. salmonis* growth in a medium without ammonium sulphate addition (BM3) to avoid a possible growth inhibition caused by ammonia accumulation. These experiments showed a retarded cell growth in propagation flasks (inoculated directly with freshly thaw cells) compared to cultures grown in BM2 (data not shown). However, no differences in growth profiles were observed when cells from the propagation flasks grown in BM3 were subcultured in fresh BM3 media. The final biomass concentration reached in these experiences was approximately 0.8 OD_600_ (SD ±0.019) after 59 h. In addition, identical pH value was recorded at the end of the cultures, reaching a value of 7.2 in both media. The ammonia concentrations in the cultures increased from approximately 63 to 105 mM in BM2 and from 0 to 48 mM in BM3.

The results indicate that regardless of the initial ammonia concentration in the medium, comparable amounts of ammonia were produced (around 40 mM); this is also supported by the identical final pH recorded in the cultures. The amount of ammonium sulphate added in the medium (BM2) was not inhibitory for growth, since as mentioned before, similar growth profiles were obtained in the experiences with BM2 and BM3. Nevertheless, since the nitrogen in the medium provided by the ammonium sulphate resulted unnecessary, it was decided to continue the research work with BM3. However, BM2 medium was always used for the propagation flasks as the cells resulted somehow sensitive to growth in BM3 when just thawed.

### Factorial design of experiments

**Figure 1:**
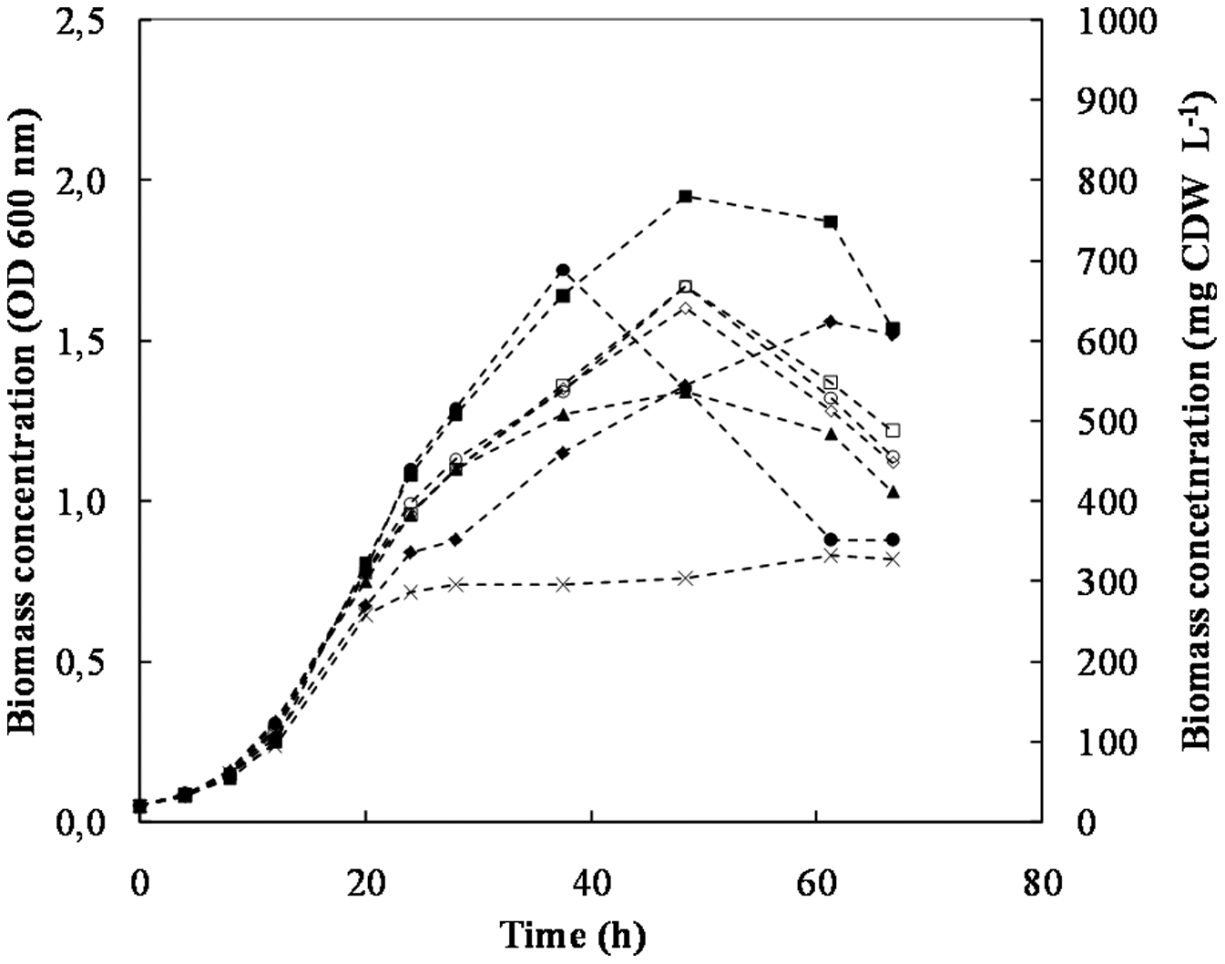
*P. salmonis* growth profiles for factorial design experiences carried out at 23°C and 100 rpm. The centre point (CP) carried out in triplicate are represented by the hollowed markers as follows: empty square (CP1), empty circle (CP2) and empty rhomb (CP3). The experiences were performed with the following concentrations of yeast extract and peptone g L^−1^, shown in brackets: CP's (6-6), filled square (8-8), filled circle (4-8), filled rhomb (8-4), filled triangle (4-4). The Control culture carried out with 2 g L^−1^ of yeast extract and 2 g L^−1^ of peptone is represented by (×).

A 2-level full factorial design of experiments was used to evaluate the significance of yeast extract and peptone on cell growth (Table 2). The results are presented in Figure 1.

The experimental error was calculated from the results of the centre point cultures performed in triplicates (CP1–CP3), which showed high reproducibility with a maximum SD of ±0.05 for cell concentration at the end of the cultures. In addition, a culture defined as control carried out in BM3 (containing 2 g L^−1^ of both, yeast extract and peptone), was performed simultaneously with the factorial design experiments for comparison purposes.

The results showed that the different yeast extract and peptone concentrations added to BM3 had a significant effect on *P. salmonis* growth, being more pronounced after 20 h of culture. It was clearly observed that the experiences performed with peptone concentration at its highest level (8 g L^−1^), were capable to reach a higher biomass concentration. The maximum volumetric productivities of biomass (Qx) as well as the maximum specific growth rates (μ_max_) are presented in Table 4.

**Table 4:** Maximum volumetric productivities (Q_x_) and growth rates (μ_max_).

Run	Yeast extract [g L^−1^]	Peptone [g L^−1^]	μ_max_ (h^−1^)	Q_x_ (mg L^−1^ h^−1^)
1	8	8	0.15	17.8
2	8	4	0.13	13.9
3	4	8	0.14	18.2
4	4	4	0.13	15.8
CP1^a^^,^ b	6	6	0.14	15.9
CP2^a^^,^ b	6	6	0.14	16.4
CP3^a^^,^ b	6	6	0.14	15.9
Control	2	2	0.12	12.7

a SD of centre points for Qx = 0.31.

b SD of centre points for μ_max_ = 0.00.

All experiments reached the maximum biomass productivity (Qx) at 24 h, considering the calculated SD (±0.31). The highest productivity was obtained in run 1 and 3 (approximately 18 mg CDW L^−1^ h^−1^), confirming the advantage of having a higher peptone concentration in the media. The lowest productivity was recorded in the control run (12.7 mg CDW L^−1^ h^−1^), presenting a value almost 30% lower than the maximum Qx above mentioned. The maximum specific growth rates were similar for all the runs and major differences in growth rates were only observed after 20 h, resulting in different final biomass concentrations as observed in Figure 1.

Furthermore, as mentioned earlier, one of the availables growth data for pure *P. salmonis* culture in liquid medium reported a maximum biomass concentration of 0.25 OD after 13 days [8]. In the present work, all the experiments exceeded by far the maximum biomass concentration described, however now after only 15 h. The runs that reached the highest biomass concentration at 37.5 h were run 1 and 3, reaching a value OD_600_ of 1.7 (670 mg CDW L^−1^), the same OD value obtained with Austral-SRS broth in 6 days [14]. Thereafter, a drastic biomass decrease was observed in run 3, while growth in run 1 was able to continue until a maximum biomass concentration of an OD_600_ value of 1.95 (776 mg CDW L^−1^) was reached after approximately 48 h, presenting later also a decrease. Likewise, after approximately 50 h a biomass concentration decrease was observed in all the runs, except in the control culture. Interestingly, a difference in pH was noticed, being around 8.0 for the factorial design experiments and 7.2 for the control. These results could indicate that *P. salmonis* growth may be sensitive to a more alkaline pH. The latter, added to a possible nutrient depletion could have contributed to the biomass concentration decrease observed. The earlier biomass concentration observed in run 3 can however not be explained at this moment.

In summary, peptone concentration appears to be more significant than the yeast extract concentration since both cultures that contained the high level of peptone (8 g L^−1^) reached the highest biomass concentration. From an industrial point of view, one of the most important process parameters is the biomass volumetric productivity (Qx). The results for the performed ANOVA, considering Qx as response (reached at 24 h of culture), indicated the peptone concentration as the only significant factor (p = 0.008). The modified BM3 medium (Run 3) that produced the high volumetric productivity was called BM4.

Additionally, in all the experiments the cultures were tested with SRS Fluorotest kit demonstrating the non-contamination with other bacterial agents (data not shown).

### *P. salmonis* growing in cell free media preserve its pathogenic potential

**Figure 2:**
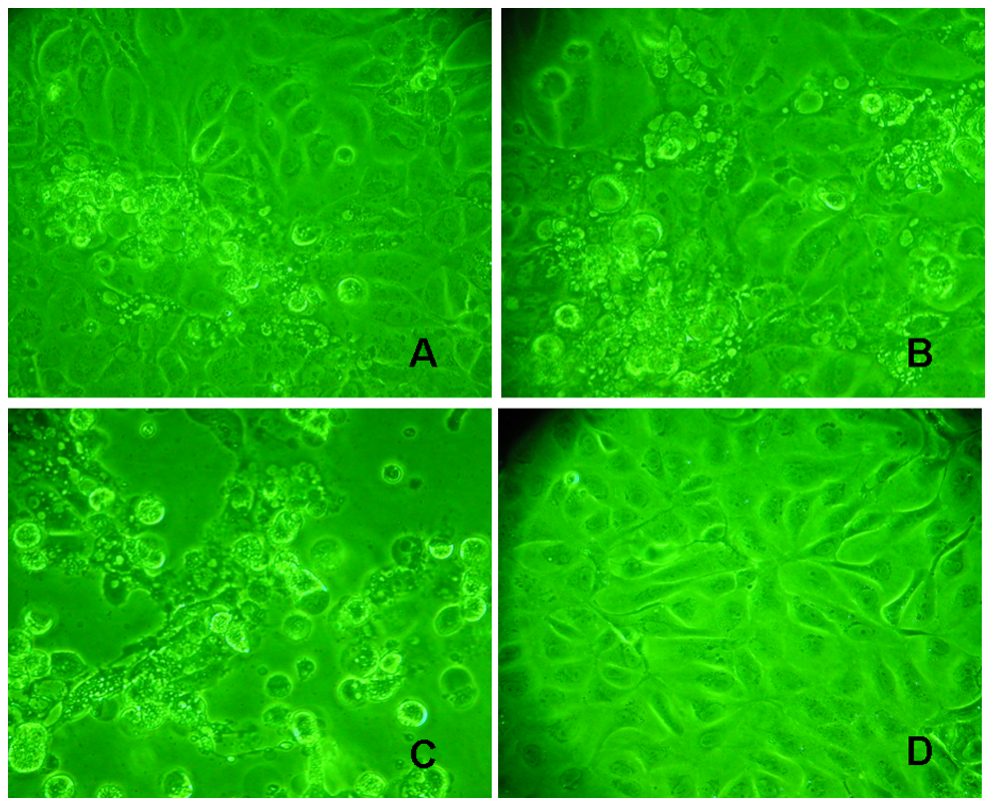
CHSE-214 cell line infected with *P. salmonis* grown in cell free media (BM1). The typical CFP produced by *P. salmonis* could be observed in all the times post-infection showing evident cell destruction after 6 days. A: 4 days post-infection; B: 6 days post-infection; C: 8 days post-infection; D: non infected cells (Control).

In order to determine the infectivity of *P. salmonis* grown in cell free media (BM4) both CHSE-214 and RTS11 cell lines were infected. In the CHSE-214 cell line the CPE start to be evident at 4 days post infection (Figure 2-A), which is characterized by the formation of clusters of rounded and vacuolized cells that eventually cause cell lysis with detach of the monolayer [11]. Cell lysis was observed after 6 days post-infection in CHSE-214 cell line (Figure 2-B and C).

**Figure 3:**
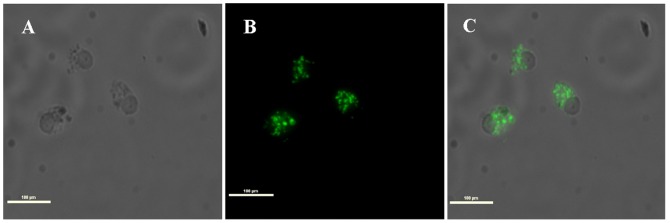
Immunofluorescence analysis of RTS11 cell line infected with *P. salmonis* grown in cell free medium (BM1). The figure shows bacteria inside the cells after 5 days post-infection. A: phase-contrast microscopy of RTS11 cell line infected with *P. salmonis*; B: Immunofluorescence of A; C: Merge of A and B. The white bar indicates 100 µm. The images were digitalized using the 40× objective.

The RTS11 cell line has two cellular populations, adherent macrophages forming a monolayer and rounded monocytes growing in suspension [18], which did not produce a CPE after *P. salmonis* infections. In order to track the infection, an immunofluorescence test was performed in this cell line after 5 days of infection, finding that the bacterium is able to infect macrophages, detecting *P. salmonis* inside these cells (Figure 3). Additionally, 15 days post-infection the cell cultures were completely lysate (data not shown).

Our results reveal that *P. salmonis* remains pathogenic *in vitro* after growing in cell free media; recently, similar results were obtained with Austral-SRS Broth [14] thus confirming our results.

## Conclusions

In conclusion, the results obtained from the initial blood free medium experiments demonstrated that *P. salmonis* is able to grow in a simple bacteriological medium and that blood or FBS constituents are not essential for the microorganism growth. Throughout this work, a three-fold increase on the maximum specific growth rate was achieved, that in turn allowed to reach a final biomass concentration, just in two days, eight times higher than previously reported in the presence of blood components [8]. On the other hand, during the factorial experiments we obtained an OD_600_ value of 1.7 only in 37.5 h, a third part of the time reported previously in the presence of FBS components and others growth factors (aminoacids and vitamins) in the culture medium [14]. Consequently, this work results present a significant advance regarding *P. salmonis* research. In addition, this is the first report that incorporates a process development perspective on the analysis of *P. salmonis* growth, with emphasis on determining improved growth conditions.

Undoubtedly, the results obtained from this study indicate that there is an enormous potential for optimizing pure *P. salmonis* growth in a liquid medium completely free of blood or FBS constituents. These results are very important, considering the strict regulatory aspects within the pharmacological industry, as well as the increase in production costs resulting from the use of a blood containing media in an industrial process.Indeed, our optimized mass production of the bacteria opens promising possibilities to generate more efficient vaccines and appears as a natural and relevant projection of our work. In fact, although at present there are several injectable vaccines against *P. salmonis* available in the market; they produce disappointing and variable long-term results. This is thought to be the result of a limited bacterial mass to dose ratio, which can be overcome with our production scheme. Additionally, the availability of adequate amounts of biomass bacteria, would also allow designing strategies to generate attenuated forms of the bacteria able to provide immunological protection far beyond the scope of action of those available today. They are effective in preventing the initial SRS outbreaks that typically occur after the transfer of salmonid fish from fresh water to sea water but not when fish become susceptible to a second more aggressive SRS outbreak affecting large fish ten to twelve months after transfer resulting in greater financial losses. Thus, protecting these fish by re-vaccination with a novel vaccine appears as an attractive solution.
